# Venous Dissection Visible on Contrast-Enhanced Computed Tomography: A Complication Associated With Replacement of a Central Venous Catheter

**DOI:** 10.7759/cureus.103246

**Published:** 2026-02-08

**Authors:** Yui Hanabusa, Aiko Suzuki, Kaoru Sumida, Masaya Ishii, Izuru Matsuda

**Affiliations:** 1 Department of Radiology, Kanto Rosai Hospital, Kawasaki, JPN

**Keywords:** central venous catheter, computed tomography, internal jugular vein, interventional radiology, venous dissection

## Abstract

Venous dissection is an uncommon complication that can occur following venous puncture, catheter insertion, or manipulation. We report a case of venous dissection that occurred during the replacement of a central venous catheter (CVC). A 77-year-old woman underwent replacement of a cuffed catheter via the left internal jugular vein. Advancement of the guidewire and dilator resulted in a venous dissection extending from the left internal jugular vein to the brachiocephalic vein, which was diagnosed upon contrast-enhanced computed tomography (CT). No further treatment was necessary, and a follow-up contrast-enhanced CT three weeks later showed partial resolution of the dissection. This case represents a rare instance in which venous dissection was successfully diagnosed using contrast-enhanced CT. Most reported cases of venous dissection have not required additional treatment. When the false lumen is patent, contrast-enhanced CT can be useful for the diagnosis of venous dissection and may help avoid unnecessary interventions.

## Introduction

Central venous catheters (CVCs) are used for total parenteral nutrition, reliable administration of medications, central venous pressure monitoring, and dialysis. Recently, CVCs have been placed mostly through the internal jugular vein. Complications associated with catheter insertion include arterial puncture, pneumothorax, hemothorax, brachial plexus injury, and air embolism [1]. Although rare, venous dissection has also been reported [1-4].

Venous dissection is thought to occur when mechanical injury to the venous intima allows blood to enter and separate the vessel wall layers, most commonly as a result of trauma, including vascular puncture [5]; however, its incidence is unknown. Identification of a flap separating the true and false lumens is essential for the diagnosis of venous dissection [6]. Previous reports have described venous dissection diagnosed during procedures using ultrasonography or angiography, and most cases did not require therapeutic intervention [1-6].

We present a case of venous dissection occurring during replacement of a cuffed catheter in the internal jugular vein, diagnosed by contrast-enhanced computed tomography (CT).

## Case presentation

A 77-year-old woman with a history of renal sclerosis, hypertension, cerebral infarction, and ureteral stones had initiated hemodialysis for renal failure. She initially had a cuffed catheter placed in the right internal jugular vein for dialysis. However, after approximately two months, it became inadequate for blood drainage. As the right internal jugular vein was occluded, a cuffed catheter was placed in the left internal jugular vein. Two weeks later, the blood flow through the catheter was again insufficient, prompting a plan for replacement.

**Figure 1:**
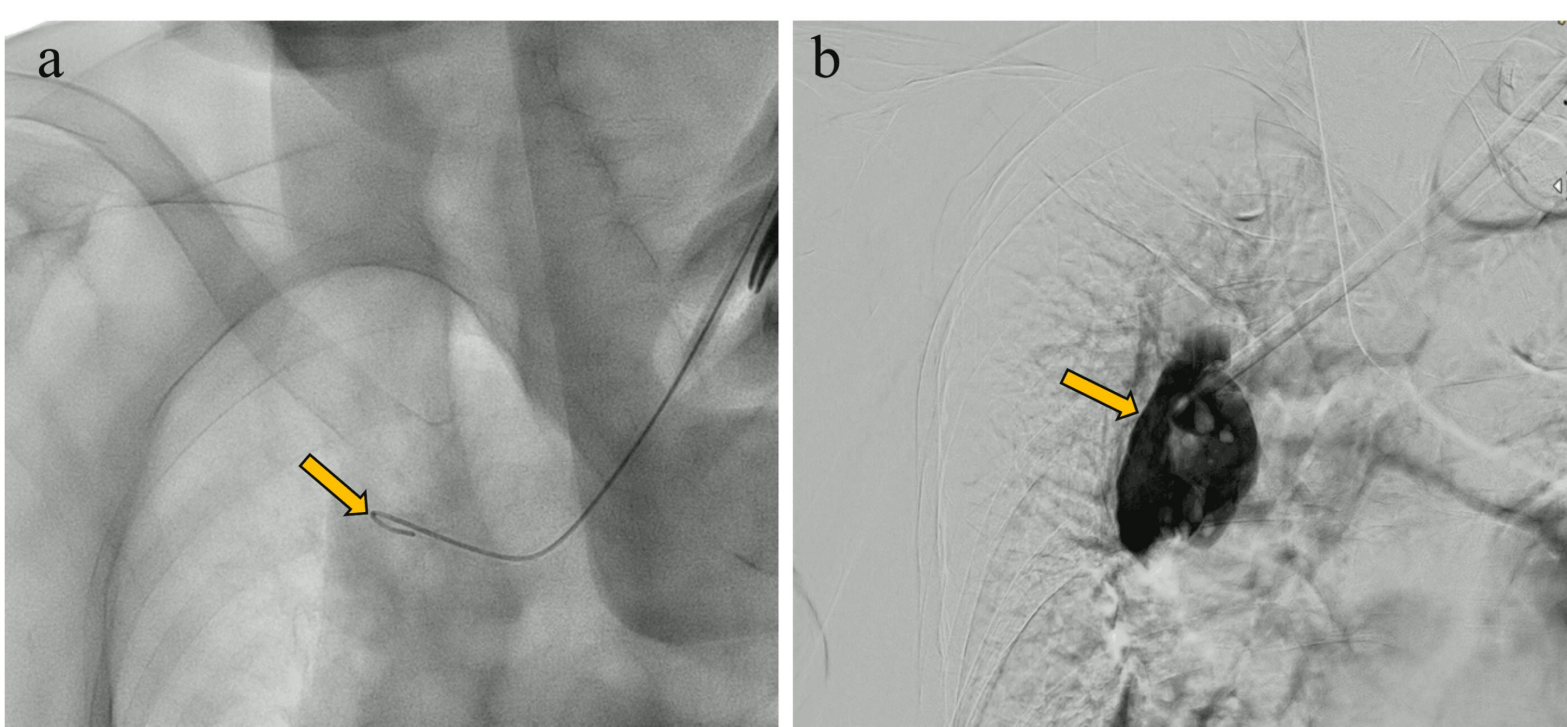
(a) After the dilator was inserted, fluoroscopy showed the guidewire (arrow) had been pulled back. (b) Contrast medium injected through the dilator leaked out of the vessel (arrow).

A 14.5-French (Fr) cuffed catheter (GlidePath; Medikon, Ankara, Turkey) was inserted in the catheterization laboratory. The surgeon inserted a 0.038-inch guidewire through the disconnected catheter into the inferior vena cava and left the guidewire in place while removing the catheter. No fluoroscopy was performed during removal. An 8-Fr dilator was inserted along the guidewire, after which fluoroscopy revealed that the guidewire had returned to a position thought to be the left brachiocephalic vein or the superior vena cava (Figure 1a). Despite manipulation of the wire and dilator, the guidewire could not be advanced into the inferior vena cava, and the patient complained of severe shoulder pain. The guidewire was removed, and contrast medium was injected through the dilator, and it leaked out of the vessel (Figure 1b).

**Figure 2:**
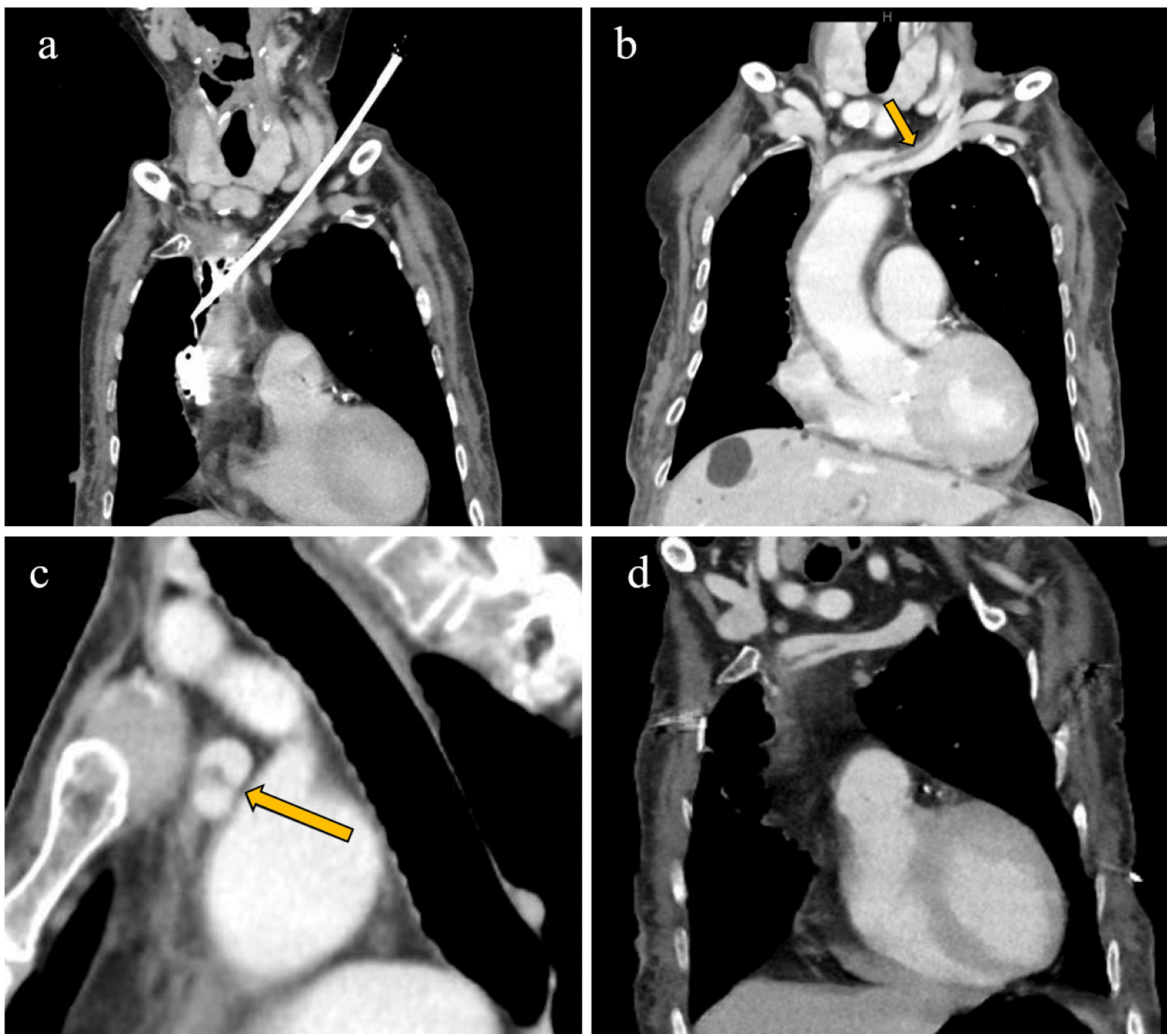
(a) CT showed the tip of the dilator outside the left brachiocephalic vein, and contrast medium had leaked into the mediastinum. (b) Contrast-enhanced CT showed the flap (arrow) and false lumen running from the proximal part of the left internal jugular vein to the brachiocephalic vein. (c) Sagittal reconstruction of contrast-enhanced CT (b) showed the flap (arrow) and false lumen in the left brachiocephalic vein. (d) Three weeks later, the flap remained, although some parts had become less distinct. CT: computed tomography

The procedure was then stopped. CT scan revealed that the tip of the dilator was positioned outside the left brachiocephalic vein, with contrast media pooling in the mediastinum (Figure 2a). Consultations with cardiovascular surgery and radiology were conducted, and the dilator was removed. The patient was transferred to the ICU after device removal, with stable vital signs.

The following morning, contrast-enhanced CT showed a flap and patent false lumen running from the proximal part of the left internal jugular vein to the brachiocephalic vein, indicating venous dissection (Figures 2b-2c). No bleeding or hematoma was observed at the site of dilator removal.

The patient did not require additional treatment and was transferred to a general ward. Ultrasonography performed at the bedside several days later could not visualize the dissection because of its deep location. Hemodialysis was performed either by direct puncture or through a cuffed catheter placed in the right femoral vein. Non-contrast CT performed 17 days after dissection showed no contrast medium or hematoma in the mediastinum and no significant dilation or constriction of the vessels from the left internal jugular vein to the brachiocephalic vein. Three weeks later, contrast-enhanced CT showed that the flap remained, but some parts became less distinct, and vasodilation improved (Figure 2d). During follow-up, the patient developed a cerebral infarction that worsened her condition, and no subsequent imaging was performed.

## Discussion

The venous wall is composed of three layers - the intima, media, and tunica externa - similar to the arterial wall [1,5-8]. Dissection occurs when the intima and media rupture, allowing blood to enter the vessel wall. There are fewer reports of venous dissection than arterial dissection, and its incidence remains unclear. This may be because the venous wall is thinner and more prone to perforation, and the venous pressure is generally lower, making it less likely for blood to flow into the vessel wall [1,6].

The most common cause of venous dissection is trauma, including puncture, though cases caused by inflammation or occurrence have also been reported [5,6]. Many venous dissections do not require additional treatment; however, some cases have been managed with antithrombotic therapy or stent placement [1-6]. Symptoms of venous dissection include pain or swelling at the dissection site and venous congestion caused by narrowing of the true lumen [5,6,8]. It can be diagnosed using ultrasonography during procedures or by angiography and CT [5]; however, some cases may go unrecognized due to their typically mild symptoms.

In this case, the guidewire appeared to have been pulled back during catheter removal without fluoroscopic guidance, and the intimal and medial layers of the vein were damaged by the dilator or guidewire during its insertion. The initial CT scan, performed while the dilator was still in place, made detailed observations of the left internal jugular and brachiocephalic veins difficult. However, contrast-enhanced CT performed after device removal revealed a flap and false lumen filled with contrast medium, confirming venous dissection.

Follow-up contrast-enhanced CT performed three weeks later showed a reduction in the vessel diameter, but the flap and false lumen persisted. Some previous reports have described the disappearance of the false lumen within 1-2 weeks, while others have observed that it remains open for two weeks or even longer [4,8]. In our case, the dissection was caused by the guidewire and dilator, and the defect in the venous wall may have been large, suggesting that the false lumen may have remained open without thrombosis during the early period. Subsequent follow-up with contrast-enhanced CT was not performed; therefore, it was unclear for how long the false lumen remained open, but our patient's course aligns with the reported tendency for the false lumen to persist without requiring intervention.

As many venous dissections do not require treatment, correct interpretation of imaging findings is essential to avoid unnecessary interventions, emphasizing the clinical value of timely imaging and accurate diagnosis. If the false lumen had completely thrombosed, distinguishing it from a venous thrombus could have been difficult; however, during the period in which it remained patent, the dissection could likely have been detected by contrast-enhanced CT.

## Conclusions

Venous dissection is an uncommon complication that can occur during procedures such as vascular puncture or catheter insertion. Although it often does not require additional treatment, careful device manipulation is recommended to reduce the risk of its occurrence. Early diagnosis is important for differentiating venous dissection from venous thrombosis or stenosis and for avoiding unnecessary interventions. In addition to ultrasonography and angiography, contrast-enhanced CT can be useful for the diagnosis of venous dissection, particularly when the false lumen remains patent, thereby facilitating appropriate clinical management.
